# Development and Assessment of Artificial Intelligence-Empowered Gait Monitoring System Using Single Inertial Sensor

**DOI:** 10.3390/s24185998

**Published:** 2024-09-16

**Authors:** Jie Zhou, Qian Mao, Fan Yang, Jun Zhang, Menghan Shi, Zilin Hu

**Affiliations:** 1 School of Apparel and Art Design, Xi’an Polytechnic University, No. 19 Jinhua South Road, Xi’an 710048, China; zhoujie@xpu.edu.cn; 2 School of Design, The Hong Kong Polytechnic University, Hong Kong, China; 3 Department of Electrical and Electronic Engineering, The Hong Kong Polytechnic University, Hong Kong, China; 4 School of Fashion and Textiles, The Hong Kong Polytechnic University, Hong Kong, China; 5 Lancaster Imagination Lab, Lancashire, Lancaster LA1 4YD, UK; m.shi2@lancaster.ac.uk; 6 School of Design, South China University of Technology, Guangzhou 510641, China; 202030101058@mail.scut.edu.cn

**Keywords:** gait monitoring, sensor, wearable system, healthcare, artificial intelligence algorithm

## Abstract

Gait instability is critical in medicine and healthcare, as it has associations with balance disorder and physical impairment. With the development of sensor technology, despite the fact that numerous wearable gait detection and recognition systems have been designed to monitor users’ gait patterns, they commonly spend a lot of time and effort to extract gait metrics from signal data. This study aims to design an artificial intelligence-empowered and economic-friendly gait monitoring system. A pair of intelligent shoes with a single inertial sensor and a smartphone application were developed as a gait monitoring system to detect users’ gait cycle, stand phase time, swing phase time, stride length, and foot clearance. We recruited 30 participants (24.09 ± 1.89 years) to collect gait data and used the Vicon motion capture system to verify the accuracy of the gait metrics. The results show that the gait monitoring system performs better on the assessment of the gait metrics. The accuracy of stride length and foot clearance is 96.17% and 92.07%, respectively. The artificial intelligence-empowered gait monitoring system holds promising potential for improving gait analysis and monitoring in the medical and healthcare fields.

## 1. Introduction

Walking is an essential activity in human daily life, and gait parameters are commonly used to diagnose clinical diseases and are suggested as critical factors in assessing human mobility [1]. Temporal and spatial gait parameters, in particular, are widely used to evaluate gait impairments caused by aging, physical injury, and neurological disorders, such as cerebellar ataxia, Alzheimer’s disease, and Parkinson’s disease [2,3]. For example, gait speed is normally suggested as a significant indicator to estimate future health status and mobility [4]. Moreover, gait variability relating to the change in stride-to-stride fluctuations is verified to be more effective than gait speed in indicating gait impairment [5]. In the investigation of fall risk among older adults, foot clearance is used as an essential indicator to predict fall risk [6,7]. Insufficient foot clearance is the cause of tripping in daily walking and going up and down stairs [8]. Except for pathological diagnosis and health management, gait parameters are also widely analyzed in exercise training. The force features of foot–ground contact are commonly used to deepen the understanding of the difference between Tai Chi Chuan and normal gait patterns, thereby prompting the application of Tai Chi Chuan in rehabilitation and fall prevention [9]. Therefore, understanding the real-time gait pattern can help monitor physical health and prevent falls.

Gait monitoring systems have evolved significantly over the past few decades, with various methods being developed to capture gait parameters in different environments. Traditional gait monitoring systems, such as optical motion capture systems and force plates [10,11], are considered gold standards for gait analysis. However, due to the need for expensive equipment and controlled conditions, they are limited to laboratory settings and are not available for natural gait monitoring during daily activities [12]. Although video-based gait monitoring systems offer a non-invasive alternative for daily motion tracking [13], they are commonly affected by external factors like lighting and camera angles. With the advances in microelectronics, wireless communication, and flexible sensor technology [14,15], wearable sensors, such as accelerometers [12], gyroscopes [16], and flexible strain sensors [17], allow for portable, real-time gait monitoring in daily activities. Particularly, inertial sensors, including accelerometers and gyroscopes, are widely integrated into smart garment-based gait monitoring systems to enable the continuous tracking of gait parameters like foot clearance and stride length [18]. However, inertial sensor-based gait monitoring systems typically estimate displacement-related gait parameters using the double integration of acceleration signals [19], leading to cumulative errors and orientation inaccuracies. These errors contribute to significant discrepancies in spatial gait parameters like stride length, walking width, and foot clearance [20,21,22].

To improve the accuracy of spatial gait parameters, numerous studies have proposed different filters to reduce drift in inertial sensor signals [23,24,25]. Moreover, miniature range sensors without accumulative error were combined with inertial sensors to obtain better performance in foot clearance estimation [26,27,28]. Despite the effectiveness of the above methods, they often rely on a flat floor assumption and increase system complexity, limiting their practical application. Recently, artificial intelligence (AI) algorithms, particularly extreme learning machines (ELMs), have been combined with inertial sensors to diagnose cerebral palsy gait and recognize biometric gait patterns [29,30]. Although the ELM algorithm presents potential in gait classification, its performance in gait monitoring systems remains uncertain.

In this context, we aimed to introduce the ELM algorithm and develop an artificial intelligence-empowered gait monitoring system with a single inertial sensor. In detail, the system calculated gait parameters (e.g., stand phase time (TSP), swing phase time (TSW), gait cycle (T), stride length, and foot clearance) from raw collected data. The ELM algorithm was then used to improve the accuracy of the calculated parameters. The feasibility of the system was evaluated by the Vicon motion capture system. The proposed gait monitoring system provide a non-invasive, wearable, and seamless solution for daily gait monitoring, leveraging people’s natural clothing habits to provide continuous, unobtrusive motion tracking.

## 2. Methods

### 2.1. Gait Monitoring System Design

**Figure 1:**
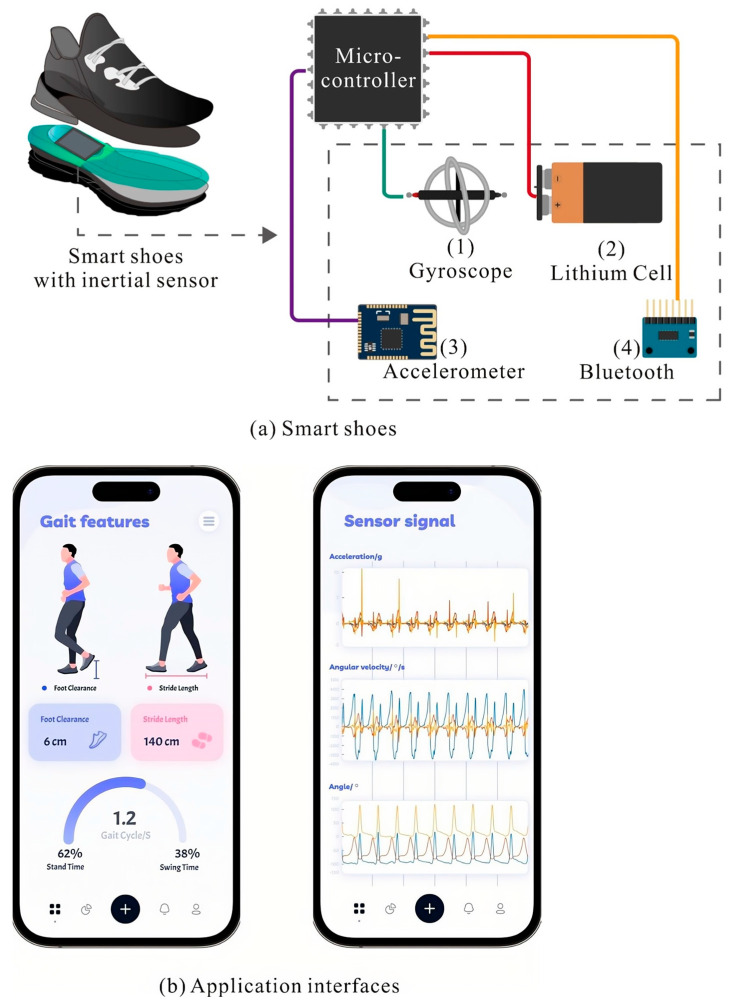
The design of the gait monitoring system: (**a**) smart shoes; (**b**) application interfaces.

In previous investigations of gait analysis, scholars have tried to attach sensors to the torso, waist, legs, instep, ankle, and so on [31]. Most sensors were put on the lower body or feet. In this study, we embedded the inertial sensor into the heel position of shoes to capture gait motion and foot clearance (see Figure 1). Since sports shoes have the function of shock absorption, support, and protection, they are reasonable for daily exercise and long-time walking. Therefore, this study focuses on sports shoes as the object to develop the gait monitoring system [32], as shown in Figure 1a. The axial direction of the sensor was perpendicular to the ground [31].

The data collection part of the hardware system was integrated with a microcomputer, Bluetooth, and an MPU-6050 sensor, including a three-axis accelerometer, a three-axis gyroscope, and other signal feedback electronic components. According to the requirements of the user manual of the sensor and Bluetooth, a 3.7V lithium battery was used to provide power for the hardware system. Additionally, we also developed application interfaces to present the analyzed gait metrics and signals, as shown in Figure 1b.

### 2.2. Evaluation Experiment

#### 2.2.1. Three-Dimensional Motion Capture Setting

**Figure 2:**
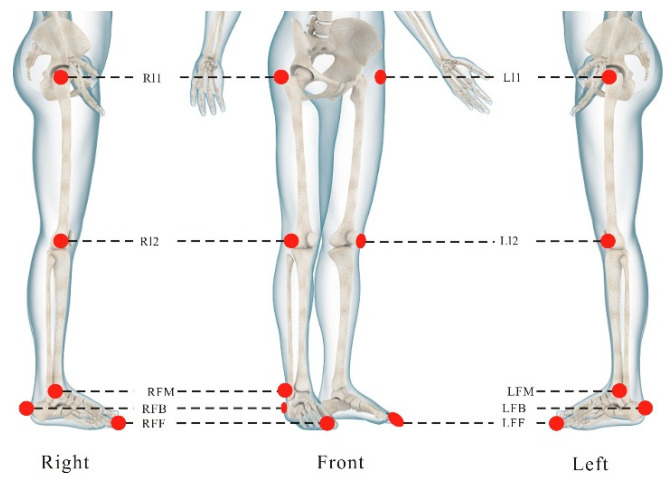
The position of the markers.

The Vicon optical motion capture system (Oxford, Oxford Metric, UK) was used in this experiment to collect the lower body motion data and evaluate the accuracy of the gait monitoring system. According to the experimental requirements and gait parameters, ten reflective markers were pasted on participants’ bodies, and the definition and illustration of the ten points are shown in Table 1 and Figure 2, respectively.

Among the ten marker points, RFB/LFB and RFF/LFF are important points for the division of gait phases. The other marker points represent the lower limb nodes, which are the keys to establishing the lower limb model.

#### 2.2.2. Subjects and Data Collection

Thirty participants (24.09 ± 1.89 years, female = 15) were recruited in this experiment. All participants involved in this study signed written informed consent forms. This experiment was carried out in a quiet, windless laboratory, with a temperature of 25 ± 2 °C and relative humidity of 65 ± 3%. The subjects were asked to walk on a treadmill with tight-fitting athletic pants. Before the formal experiment, the participants were asked to walk on the treadmill for 3 min according to their walking habits. Then, a professional tester pasted high-reflection markers on the surface of their body according to Table 2. The Vicon system was calibrated before this experiment began. Finally, the Vicon motion capture system and gait monitoring system were used to collect gait data synchronously. Firstly, the subjects stood naturally on a treadmill, and the static data of 10 s were collected. After collecting the static data, one minute of walking data was collected. Each experiment was conducted 3 times with an interval of 5 min.

The proposed gait monitoring system collected the raw data of triaxial (anterior–posterior direction, medio-lateral direction, vertical direction) acceleration, angle, and angular velocity, while the Vicon optical motion capture system recorded the triaxial coordinate of ten markers.

### 2.3. Data Processing

The relative limb angle (RLA) method was used to define the gait cycle in this research. A complete gait cycle was defined as the time period from the initial contact of the left foot to the next initial contact of the left foot. Before the division of the gait cycles, the original data were tested for missing values and outliers. For the Vicon system, marker points may be blocked by the treadmill and limbs during human motion, and this fact results in the abnormality and lack of coordinate data. While there are no missing phenomena in the signals of the smart shoes, there are some abnormal data among them. These data will cause a large error if they are used to estimate the gait parameters directly.

Missing values and outliers were tested for the coordinate data of lower limbs collected by Vicon. Herein, the amplitude of acceleration, angular velocity, and joint angle was used to analyze the outlier of the signals obtained from the smart shoes. The amplitude that was smaller than the first quartile (1.5interquartile range) or larger than the third quartile + (1.5interquartile range) were considered as outliers [32]. Gait cycles with missing values and outliers were simultaneously deleted from the data collected by Vicon and smart shoes. A total of 54 gait cycles were analyzed for each subject, and a total of 1620 gait cycles were gained.

### 2.4. Prediction Model

**Figure 3:**
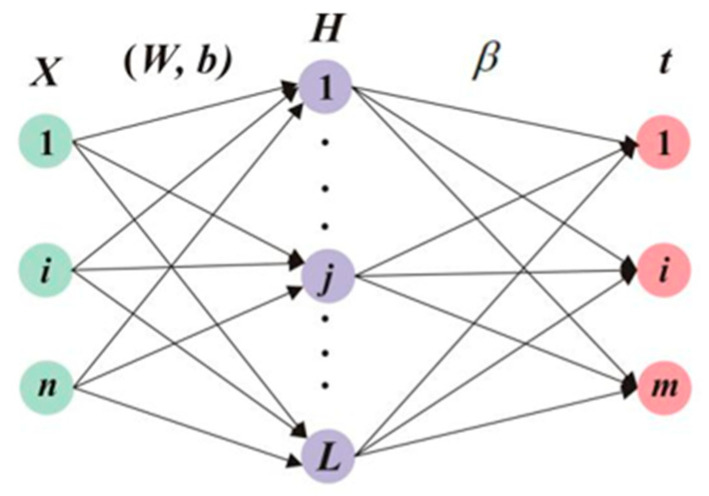
ELM topological structure.

Recently, artificial intelligence algorithms have been widely used for human motion prediction [33,34]. With reasonable training, neural networks have the ability to obtain real-time simulation and estimation for complex human actions like gait. For the issue of reduced generalization performance and overtraining in the conventional feed-forward neural network, Huang et al. proposed an extreme learning machine algorithm (ELM) based on Moor–Penrose (MP) generalized inverse matrix theory [35], and its topological structure is shown in Figure 3. In this algorithm, input layer weight and hidden layer bias are randomly selected, and only two hidden layer parameters (activation function and the number of neurons) are set during operation to obtain the optimal solution. This method has the advantages of fewer training parameters, strong generalization ability, and fast learning speed, so it is widely used in data prediction and recognition in multiple fields.

Suppose the training set sample is (*X_i_*, *t_i_*), $X_{i}={\left[x_{i1},\;x_{i2},\;\cdots x_{in}\right]}^{T}\in R^{n}$, and $t_{i}={\left[t_{i1},\;t_{i2},\;\cdots t_{im}\right]}^{T}\in R^{m}$. The equation of a single-hidden-layer neural network (hidden layer node is L) is expressed as

(1) $$\sum_{i=1}^{L}\beta_{i}g\left(W_{i}\cdot X_{j}+b_{i}\right)=o_{j},j=1,\cdots ,N$$

where bi is the bias of the i hidden layer unit; $\beta_{i}$ is the output weight; g(x) represents the activation function, usually including sig, sin, hardlim, and three other functions Hβ=T [35]. $W_{i}={\left[w_{i,1},\;w_{i,2},\;\cdots w_{i,n}\right]}^{T}$ is the input weight; $W_{i}\cdot X_{i}$ indicates the inner product of Wi and Xi.

In order to minimize the output error of a single-hidden-layer neural network, the following is calculated:

(2) $$\sum_{j=1}^{N}\left\|o_{j}-t_{j}\right\|=0$$

where $\beta_{i}$, $W_{i}$, and $b_{i}$ make Equation (1), which can be expressed as Hβ=T. T is the expected output, H is the output of the hidden layer node, and β is the output weight:

(3) $$H\left(W_{1},\cdots W_{L},b_{1},\cdots b_{L},X_{1}\cdots X_{L}\right)={\left[\begin{array}{ccc} g\left(W_{1}\cdot X_{1}+b_{1}\right) & \cdots & g\left(W_{L}\cdot X_{1}+b_{L}\right)\\ \vdots & \cdots & \vdots \\ g\left(W_{1}\cdot X_{N}+b_{1}\right) & \cdots & g\left(W_{L}\cdot X_{N}+b_{L}\right) \end{array}\right]}_{N\times L}$$

(4) $$\beta ={\left[\begin{array}{c} \beta_{1}^{T}\\ \vdots \\ \beta_{L}^{T} \end{array}\right]}_{L\times m}T={\left[\begin{array}{c} T_{1}^{T}\\ \vdots \\ T_{L}^{T} \end{array}\right]}_{N\times m}$$

Then, $\overset{\wedge }{W_{i}}$, $\overset{\wedge }{b_{i}}$, and $\overset{\wedge }{\beta_{i}}$ are obtained for training a single-hidden-layer neural network:

(5) $$\left\|H\left({\overset{\wedge }{W}}_{i},{\overset{\wedge }{b}}_{i}\right){\overset{\wedge }{\beta }}_{i}-T\right\|=\underset{W,b,\beta }{\min}\left\|H\left(W_{i},b_{i}\right)\beta_{i}-T\right\|$$

i=1,2,⋯L, which is the Loss minimization function:

(6) $$E={\sum_{j=1}^{N}\left(\sum_{i=1}^{L}\beta_{i}g\left(W_{i}\cdot X_{j}+b_{i}\right)-t_{j}\right)}^{2}$$

According to the above calculation process, the network training of the extreme learning machine is transformed into a linear system Hβ=T [35].

**Figure 4:**
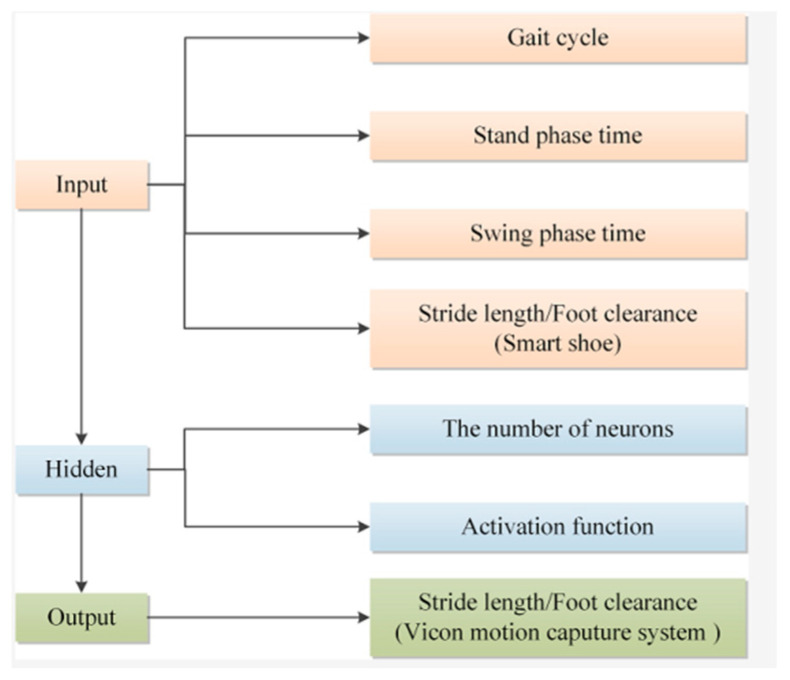
Enhanced models for gait spatial parameters.

The prediction model of gait spatial parameters was built by MATLAB R2021a based on the ELM algorithm. In this study, the spatial parameters of gait include stride length and foot clearance. Due to the large difference between the two values, prediction models were established for the two gait parameters to improve the prediction accuracy of the model, as shown in Figure 4.

Stand phase time, swing phase time, gait cycle, and stride length measured by smart shoes were the input layer of the stride length enhanced model, with a total of 4 nodes. The real stride length measured by the Vicon system was taken as the output layer. As for the number of neurons in the hidden layer and activation function of the gait parameter correction model, there are differences in parameter setting among different types of data sets. And there is no standard setting method. Therefore, the determination coefficient (*R*^2^) of the step size prediction model was taken as the evaluation index, and the trial-and-error method was adopted to obtain relatively optimal hidden layer parameters.

Firstly, according to empirical Equation (7) [36], the number of neurons in the hidden layer is 3~12:

(7) $$L=\sqrt{n+m}+a$$

where *L*, *n*, and *m* are the number of neurons in the hidden layer, input layer, and output layer, respectively. *a* is a constant between 1 and 10.

However, to improve the accuracy of the model, this study expanded the scope of the trial-and-error method. The number of neurons in the hidden layer was set as 5, and then the difference was increased to 50 to obtain the *R*^2^ of the model under the sig, sin, and hardlim activation functions.

## 3. Results and Discussion

### 3.1. Phase Division of Gait Cycle

**Figure 5:**
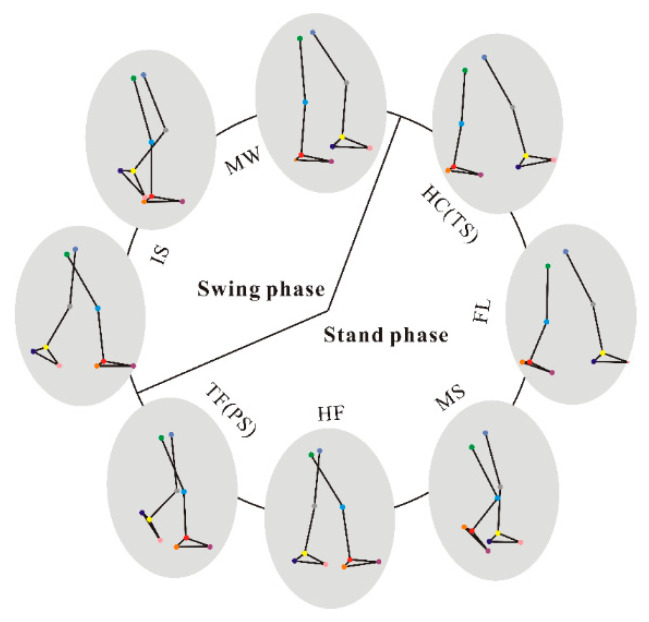
A gait cycle and lower limb features.

According to the human lower limb model captured by the Vicon system, the main features of the lower limb model in a complete gait cycle were intercepted by combining the RLA time phase division method, as shown in Figure 5.

When the gait is divided according to the motion characteristics of one leg, the heel contact (HC) event is taken as the starting point of the gait cycle, and the foot will be completely flat (FL) after a short time. Then, the weight of the body moves noticeably towards the right leg and pushes the body forward, which is called mid-standing (MS). From the occurrence of the heel lifting (HF) event, the gait begins to enter the end of the standing phase, until the occurrence of the toe off the ground (TF) event. At this time, the support phase of the gait cycle ends, and the gait enters the swing phase. The swing phase can be divided into pre-swing (PS), initial swing (IS), middle swing (MW), and terminal swing (TS). PS and TS are equivalent to the TF and HC events, while IS and MW are the processes of the swing phase. The human foot movement is accelerated during IS, while it is the opposite during MS. As far as healthy people are concerned, the gait characteristics of both feet are symmetrical [37]. Therefore, this work only focuses on the gait phase division of the left foot.

**Figure 6:**
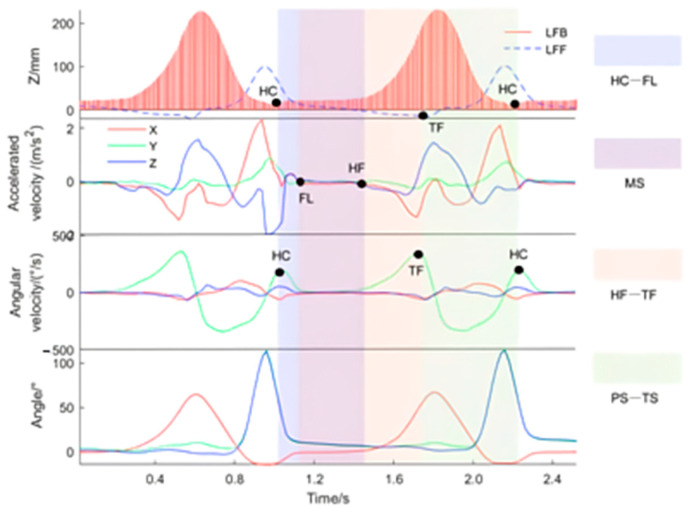
Gait cycle characteristics (X: anterior–posterior direction; Y: medio-lateral direction; Z: vertical direction).

After the phase division of gait, it is necessary to correlate this with the data signal features of the two gait acquisition systems so as to realize the extraction of the spatial–temporal parameters of gait. The gait was divided by the motion track of the heel LFB and toe LFF in the vertical direction. The characteristic point corresponding to the heel touching (HC) event is the lowest point of LFB on the Z-axis, and the characteristic point corresponding to the toe off the ground (TF) event is the lowest point of LFF on the Z-axis (see Figure 6).

The vertical motion signals of the LFB and LFF points in a gait cycle were extracted based on the Vicon system time-series data. The signal was compared with acceleration, angular velocity, and angle signals collected by smart shoes, and the gait characteristic point information contained in these three sensor signals was analyzed, as shown in Figure 6.

Figure 6 shows that acceleration signals contain more waveform noises than angular velocity and angle signals. The reason for this is that the accelerometer is more sensitive to vibration, and the shorter the impact time, the more the impact acceleration noise. Therefore, for the events that occurred in the process of foot movement, the characteristics of the acceleration curve are not obvious, and there is no clear pattern. However, the characteristics of acceleration signals during the static state of the foot are more stable than those of the other two sensors and can detect the occurred moment of FL and HF events. HC and TF events can be judged according to the extreme point characteristics of Y-axis angular velocity.

According to the motion characteristics of the feet, a complete gait can be clearly divided into four stages, which was shown as a color module in Figure 6. The first stage is from the HC event to the FL event; the characteristic points corresponding to the HC event are the lowest point of LFB in the Z-axis direction and the maximum point with a small angular velocity in the Y-axis direction. The characteristic points corresponding to the FL event are the moments when the three axial acceleration values all tend to zero. The second stage is the MS process, that is, the static state of the foot from the FL event to the HF event. The characteristic points corresponding to the HF event are the moments when the three axial acceleration values change from zero. The third stage is from the HF event to the TF event. The TF event corresponds to the lowest point of LFF in the Z-axis direction and the maximum point of angular velocity in the Y-axis direction. The first three stages all belong to the stance phase of gait. The fourth stage is the swing phase of gait, starting from the TF event to the HC event.

### 3.2. Temporal and Spatial Gait Parameters

After dividing the stance phase and swing phase, the gait cycle (*T*), stand phase time (*T_SP_*), and swing phase time (*T_SW_*) can be calculated according to the characteristic point moments of HC and TF events. Since the occurrence time of the characteristic points of gait is obtained, and events are consistent in sensor signals and Vicon system observation curves, the proposed gait monitoring system can accurately extract the gait time parameters. Suppose that the HC event and TF event occur at *t_HC_(k)* and *t_TF_(k)* in the *k* gait cycle.

Taking the HC event as the starting point of the gait cycle, the gait cycle T(k) of the k cycle is as follows:

*T*(*k*) = *t_HC_*(*k* + 1) − *t_HC_*(*k*) (8)

The *T_SP_*(*k*) of the stand phase in the *k* cycle is as follows:

*T_SP_*(*k*) = *t_TF_*(*k*) − *t_HC_*(*k*) (9)

The *T_SW_*(*k*) of the swing phase in the *k* cycle is as follows:

*T_SW_*(*k*) = *t_HC_*(*k* + 1) − *t_TF_*(*k*) (10)

After calculation, the temporal gait parameters are shown in Table 2.

The gait cycle of males is longer than that of females, and the time difference between the two is mainly reflected in the duration of the support phase. Therefore, it is speculated that the change in gait cycle is mainly caused by the stand phase, which may be because the proportion of the stand phase is relatively large, while the proportion of the swing phase is relatively small.

The stride length generally refers to the distance between the landing sites of both feet. However, since subjects who exercise on a treadmill with smart shoes only had data collected on the gait of the left foot, the movement displacement of the same foot in a gait cycle was adopted to define the stride length.

**Figure 7:**
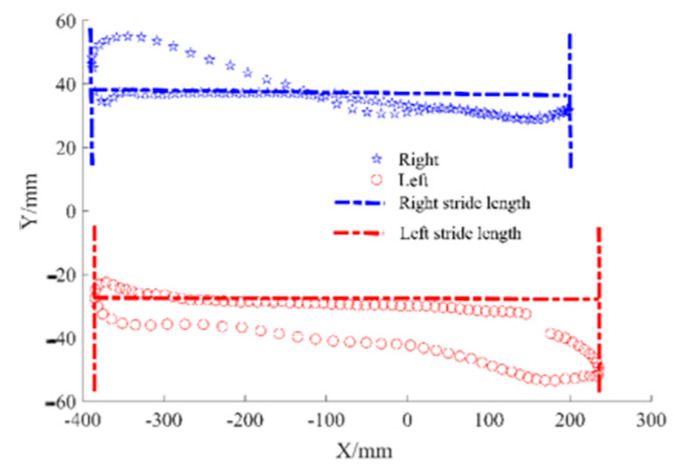
The trajectory of the feet in the X-Y plane.

The methods of stride length extraction in the two gait measurement systems are different. The data collected by the Vicon system recorded the movement track of marker points in three-dimensional space. According to the projection of LFB and RFB on the X-Y plane, the motion characteristics of the feet can be obtained, as shown in Figure 7. Since the movement on the treadmill is relative, the feet show a back-and-forth trajectory, with the length up the X-axis representing the stride length.

The stride length can be extracted from the acceleration signal of the gait monitoring system in the human body’s forward direction, and the speed variable can be obtained by integrating acceleration, and then the stride length parameter can be obtained by integrating speed. However, the velocity needs to be treated with the zero-velocity correction method before it can be integrated again, so as to obtain a more accurate displacement.

**Figure 8:**
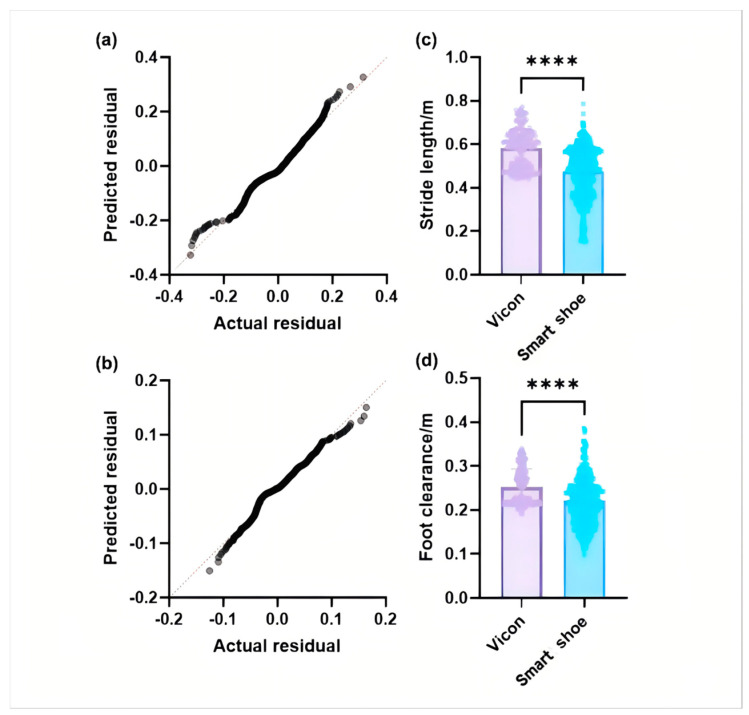
A normal distribution test and difference analysis: (**a**) a Q-Q plot of stride length; (**b**) a Q-Q plot of foot clearance; (**c**) the difference in stride length; (**d**) the difference in foot clearance. (**** in the (**c**,**d**) indicates that the *p*-value is less than 0.0001 and the difference between the two sets of data is very high).

The stride length and foot clearance are checked for normal distribution and analyzed for differences between the Vicon system and gait monitoring system. The Mann–Whitney U test is adopted to detect the difference in stride length and foot clearance. The results are shown in Figure 8.

As shown in Figure 8, the spatial gait parameters extracted from the two systems have strong differences, which indicates that the original calculated parameters of the gait monitoring system cannot achieve the accuracy of the Vicon system. It is thus necessary to employ algorithms to enhance the accuracy of the extracted stride length and foot clearance.

### 3.3. The Prediction of the Spatial Gait Parameters

The number of neurons in the hidden layer was set to five first, and then the difference was increased to 50 to obtain the *R^2^* of the model under the activation functions of sig, sin, and hardlim. The results are shown in Table 3.

As can be seen from Table 3, when the number of neurons is 40, the stride length prediction model under the three activation functions reach the maximum *R*^2^. The larger *R*^2^ is, the higher the prediction accuracy of the model is. When the activation function is ‘sig’, the performance of the stride length prediction model is relatively the best (*R*^2^ = 0.9588). Therefore, the topological structure of the prediction model is finally determined as 4-40-1, and the activation function is ‘sig’.

After the model parameters were determined, the stride length prediction model was constructed. Firstly, the training set and test set of the gait parameter prediction model were randomly selected. The sample set includes 1620 sets of data, among which the training set accounts for 94% and the test set for 6%. Then, the sample data were normalized. After that, the training set samples were sent to the network input layer and output layer to train the model. Finally, based on the trained ELM gait parameter prediction model, the simulation prediction of the test set samples was carried out.

**Figure 9:**
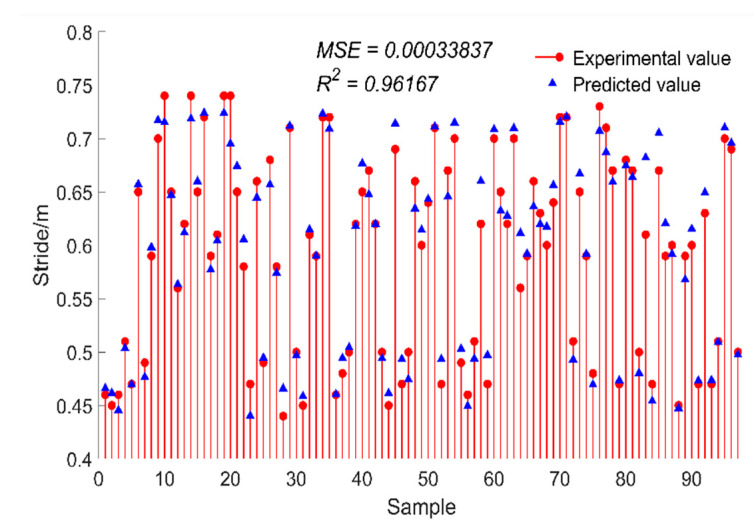
The predicted value of stride length.

**Figure 10:**
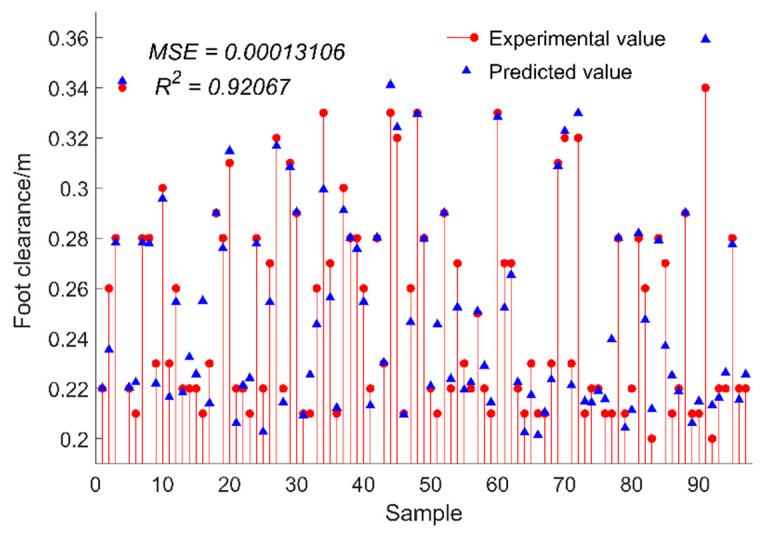
The predicted value of foot clearance.

The prediction model of foot clearance only needs to replace the stride length parameter with foot clearance, and the topological structure of the model remains unchanged. With the R2 and mean square error (MSE) as the accuracy evaluation indexes of the gait parameter prediction model, the calculation equation is as follows.

(11) $$R^{2}=1-\frac{\sum_{i=1}^{m}{\left(x^{\left(i\right)}-x^{\prime \prime (i)}\right)}^{2}}{\sum_{i=1}^{m}{\left(x^{\prime \prime (i)}-\bar{x^{\prime \prime }}\right)}^{2}}$$

(12) $$MSE=\frac{1}{m}\sum_{i=1}^{m}{\left(x^{\left(i\right)}-x^{\prime \prime (i)}\right)}^{2}$$

where m represents the number of test sets; $x^{\left(i\right)}$ represents the true value of gait parameters, which is the measured value of the Vicon system; $x^{\prime \prime (i)}$ represents the predicted values of gait parameters by the prediction model; $\bar{x^{\prime \prime }}$ represents the average predicted value of gait parameters. After calculation, the results are shown in Figure 9 and Figure 10.

As can be seen from Figure 10, for the test set sample, the evaluation index *R*^2^ of this research model reached 96.17%, and the MSE was 0.0003. Therefore, the gait parameter prediction model based on the ELM algorithm can effectively predict the real stride length according to the gait space–time parameters detected by the gait monitoring system. By observing the numerical difference between the predicted value and the real value, it can be found that the predicted value of stride length is mostly larger than the real value.

As can be seen from Figure 10, the *R*^2^ value of the test set sample is 92.07% in terms of foot clearance, 4.10% less than the stride length. However, the MSE of this model is 0.0001, so the ELM algorithm also has a good predicted effect on foot clearance. Contrary to the stride length, the predicted value of foot clearance is less than the real value.

While this study demonstrated the proposed system’s efficacy in a controlled laboratory environment, additional testing in various real-world settings (e.g., outdoor, uneven terrain, and different weather conditions) is necessary to fully validate the developed gait monitoring system’s environmental adaptability. Future work should include tests across diverse environments to assess the system’s performance under varying conditions, such as temperature, humidity, and surface types, ensuring the system’s robustness and accuracy in daily scenarios.

## 4. Conclusions

An artificial intelligence-empowered gait monitoring system was developed based on a single inertial sensor to estimate the gait parameters in this paper. To evaluate the reliability of the system, temporal and spatial gait parameters, including the gait cycle, stand phase time, swing phase time, stride length, and foot clearance, were extracted from the data collected by the Vicon motion capture system and the proposed system, respectively. It was found that the proposed system can efficiently estimate temporal parameters. Although there are some errors in the original estimation of spatial gait parameters in the proposed system, the accuracy of stride length and foot clearance can be enhanced by the established prediction model. The *R*^2^ of the prediction model based on an extreme learning machine are all more than 90%, and the MSEs are all less than 0.001. Since the temporal and spatial gait parameters can reflect gait representation in human walking and are the key indicators to detect abnormal gait, the proposed gait monitoring system has the potential to monitor and provide early warnings if an abnormal daily gait is detected.

## Figures and Tables

**Table 1:** The definitions of the marker positions.

Marker	Description	Definition	Marker	Description	Definition
LL1	Left greater trochanter	The most lateral prominence of the left greater trochanter	RL1	Right greater trochanter	The most lateral prominence of the right greater trochanter
LL2	Left lateral femoral epicondyle	The most lateral prominence of the left lateral femoral epicondyle	RL2	Right lateral femoral epicondyle	The most lateral prominence of the right lateral femoral epicondyle
LFM	Left heel	Left posterior calcaneus	RFM	Right heel	Right posterior calcaneus
LFB	Left lateral malleolus	The lateral prominence of the left lateral tibial malleolus	RFB	Right lateral malleolus	The lateral prominence of the right lateral tibial malleolus
LFF	Left 2nd metatarsal head	The dorsal aspect of the left second metatarsal head	RFF	Right 2nd metatarsal head	The dorsal aspect of the right second metatarsal head

**Table 2:** Temporal gait parameter unit: s.

Gait Cycle	Female			Male		
	*Tsp*	*Tsw*	*T*	*Tsp*	*Tsw*	*T*
1	0.81	0.40	1.21	0.85	0.41	1.26
2	0.82	0.37	1.19	0.85	0.41	1.26
…	…	…	…	…	…	…
1620	0.78	0.43	1.21	0.83	0.39	1.22
Mean	0.82	0.40	1.21	0.81	0.39	1.20
Max	0.87	0.48	1.31	0.88	0.43	1.28
Min	0.69	0.31	1.09	0.69	0.34	1.07

**Table 3:** The *R*^2^ of stride length predicted ELM model.

The Number of Neurons	sig	sin	Hardlim
5	0.7749	0.7009	0.0012
10	0.9044	0.8879	0.2328
15	0.8966	0.8730	0.2521
20	0.9168	0.8859	0.1980
25	0.9270	0.9091	0.7584
30	0.9571	0.9257	0.7559
35	0.9551	0.9403	0.6977
40	0.9588	0.9498	0.7832
45	0.9543	0.9390	0.6076
50	0.9300	0.9390	0.8244

## Data Availability

The data are available from the authors upon reasonable request.

## References

[1] Tao W., Liu T., Zheng R., Feng H. (2012). Gait analysis using wearable sensors. Sensors.

[2] Alcock L., Galna B., Lord S., Rochester L. (2016). Characterisation of foot clearance during gait in people with early Parkinson’s disease: Deficits associated with a dual task. J. Biomech..

[3] Pirker W., Katzenschlager R. (2017). Gait disorders in adults and the elderly. Wien. Klin. Wochenschr..

[4] Middleton A., Fritz S.L., Lusardi M. (2015). Walking speed: The functional vital sign. J. Aging Phys. Act..

[5] Hausdorff J.M. (2009). Gait dynamics in Parkinson’s disease: Common and distinct behavior among stride length, gait variability, and fractal-like scaling. Chaos Interdiscip. J. Nonlinear Sci..

[6] Pfortmueller C., Lindner G., Exadaktylos A. (2014). Reducing fall risk in the elderly: Risk factors and fall prevention, a systematic review. Minerva Med..

[7] Kearney F.C., Harwood R.H., Gladman J.R., Lincoln N., Masud T. (2013). The relationship between executive function and falls and gait abnormalities in older adults: A systematic review. Dement. Geriatr. Cogn. Disord..

[8] Telonio A., Blanchet S., Maganaris C., Baltzopoulos V., McFadyen B. (2013). The detailed measurement of foot clearance by young adults during stair descent. J. Biomech..

[9] Wu G., Hitt J. (2005). Ground contact characteristics of Tai Chi gait. Gait Posture.

[10] Graci V., Elliott D.B., Buckley J.G. (2009). Peripheral visual cues affect minimum-foot-clearance during overground locomotion. Gait Posture.

[11] Miyashiro K., Nagahara R., Yamamoto K., Nishijima T. (2019). Kinematics of maximal speed sprinting with different running speed, leg length, and step characteristics. Front. Sports Act. Living.

[12] Zijlstra W. (2004). Assessment of spatio-temporal parameters during unconstrained walking. Eur. J. Appl. Physiol..

[13] Mehdizadeh S., Nabavi H., Sabo A., Arora T., Iaboni A., Taati B. (2021). Concurrent validity of human pose tracking in video for measuring gait parameters in older adults: A preliminary analysis with multiple trackers, viewing angles, and walking directions. J. NeuroEngineering Rehabil..

[14] Xing T., He A., Huang Z., Luo Y., Zhang Y., Wang M., Shi Z., Ke G., Bai J., Zhao S. (2023). Silk-based flexible electronics and smart wearable Textiles: Progress and beyond. Chem. Eng. J..

[15] Hao Y., Yan Q., Liu H., He X., Zhang P., Qin X., Wang R., Sun J., Wang L., Cheng Y. (2023). A stretchable, breathable, and self-adhesive electronic skin with multimodal sensing capabilities for human-centered healthcare. Adv. Funct. Mater..

[16] Shi G., Chan C.S., Li W.J., Leung K.-S., Zou Y., Jin Y. (2009). Mobile human airbag system for fall protection using MEMS sensors and embedded SVM classifier. IEEE Sens. J..

[17] Zhang L., Zhou M., He Y., Wang L., Song H., Du H., Liu H., Liu C. (2024). Flexible porous non-woven silk fabric based conductive composite for efficient multimodal sensing. Chem. Eng. J..

[18] Scataglini S., Abran G., Roosens E., Van Tiggelen D., Haelterman R., Verwulgen S. (2020). Smart clothing for monitoring gait. DHM2020.

[19] Kitagawa N., Ogihara N. (2016). Estimation of foot trajectory during human walking by a wearable inertial measurement unit mounted to the foot. Gait Posture.

[20] Mariani B., Rochat S., Büla C.J., Aminian K. (2012). Heel and toe clearance estimation for gait analysis using wireless inertial sensors. IEEE Trans. Biomed. Eng..

[21] Hannink J., Ollenschläger M., Kluge F., Roth N., Klucken J., Eskofier B.M. (2017). Benchmarking foot trajectory estimation methods for mobile gait analysis. Sensors.

[22] van den Tillaar R., Nagahara R., Gleadhill S., Jiménez-Reyes P. (2021). Step-to-Step Kinematic Validation between an Inertial Measurement Unit (IMU) 3D System, a Combined Laser+ IMU System and Force Plates during a 50 M Sprint in a Cohort of Sprinters. Sensors.

[23] Rebula J.R., Ojeda L.V., Adamczyk P.G., Kuo A.D. (2013). Measurement of foot placement and its variability with inertial sensors. Gait Posture.

[24] Olivares A., Górriz J., Ramírez J., Olivares G. (2016). Using frequency analysis to improve the precision of human body posture algorithms based on Kalman filters. Comput. Biol. Med..

[25] Takeda R., Lisco G., Fujisawa T., Gastaldi L., Tohyama H., Tadano S. (2014). Drift removal for improving the accuracy of gait parameters using wearable sensor systems. Sensors.

[26] Arami A., Saint Raymond N., Aminian K. (2017). An accurate wearable foot clearance estimation system: Toward a real-time measurement system. IEEE Sens. J..

[27] Duong P.D., Suh Y.S. (2015). Foot pose estimation using an inertial sensor unit and two distance sensors. Sensors.

[28] Wahab Y., Bakar N., Mazalan M. (2014). Error correction for foot clearance in real-time measurement. J. Phys. Conf. Ser..

[29] Chakraborty S., Nandy A. (2020). Automatic diagnosis of cerebral palsy gait using computational intelligence techniques: A low-cost multi-sensor approach. IEEE Trans. Neural Syst. Rehabil. Eng..

[30] Patil P., Kumar K.S., Gaud N., Semwal V.B. Clinical human gait classification: Extreme learning machine approach. Proceedings of the 2019 1st International Conference on Advances in Science, Engineering and Robotics Technology (ICASERT).

[31] Storm F.A., Cesareo A., Reni G., Biffi E. (2020). Wearable inertial sensors to assess gait during the 6-minute walk test: A systematic review. Sensors.

[32] Zhou J., Mao Q., Zhang J., Lau N.M., Chen J. (2022). The relevance of breast motions and gaits in running exercises. Fash. Text..

[33] Schöllhorn W.I. (2004). Applications of artificial neural nets in clinical biomechanics. Clin. Biomech..

[34] Ezati M., Ghannadi B., McPhee J. (2019). A review of simulation methods for human movement dynamics with emphasis on gait. Multibody Syst. Dyn..

[35] Huang G.-B., Zhu Q.-Y., Siew C.-K. (2006). Extreme learning machine: Theory and applications. Neurocomputing.

[36] Jie Z., Qiurui M. (2020). Establishing a Genetic Algorithm-Back Propagation model to predict the pressure of girdles and to determine the model function. Text. Res. J..

[37] Allet L., Armand S., Golay A., Monnin D., De Bie R., de Bruin E.D. (2008). Gait characteristics of diabetic patients: A systematic review. Diabetes/Metab. Res. Rev..

